# Aging Increases Prosocial Motivation for Effort

**DOI:** 10.1177/0956797620975781

**Published:** 2021-04-16

**Authors:** Patricia L. Lockwood, Ayat Abdurahman, Anthony S. Gabay, Daniel Drew, Marin Tamm, Masud Husain, Matthew A. J. Apps

**Affiliations:** 1 Centre for Human Brain Health, School of Psychology, University of Birmingham; 2 Department of Experimental Psychology, University of Oxford; 3 Wellcome Centre for Integrative Neuroimaging, Department of Experimental Psychology, University of Oxford; 4 Christ Church, University of Oxford; 5 Department of Psychology, University of Cambridge

**Keywords:** prosocial behavior, aging, effort, motivation, reward, computational modeling, open data

## Abstract

Social cohesion relies on prosociality in increasingly aging populations. Helping other people requires effort, yet how willing people are to exert effort to benefit themselves and others, and whether such behaviors shift across the life span, is poorly understood. Using computational modeling, we tested the willingness of 95 younger adults (18–36 years old) and 92 older adults (55–84 years old) to put physical effort into self- and other-benefiting acts. Participants chose whether to work and exert force (30%–70% of maximum grip strength) for rewards (2–10 credits) accrued for themselves or, prosocially, for another. Younger adults were somewhat selfish, choosing to work more at higher effort levels for themselves, and exerted less force in prosocial work. Strikingly, compared with younger adults, older people were more willing to put in effort for others and exerted equal force for themselves and others. Increased prosociality in older people has important implications for human behavior and societal structure.

The world’s population is aging. As people age, social interactions are vital for sustaining health and well-being because social isolation is significantly detrimental to physical and mental health (Fratiglioni et al., 2004). Social cohesion depends on motivation and on people being willing to incur costs to help others (Fehr & Fischbacher, 2003). Many prosocial behaviors have been extensively studied in children and young adults (Eisenberg et al., 2005; Imuta et al., 2016), and consequently, conclusions about the boundaries of human prosociality are largely based on these populations alone. However, much less is known about them in older adults. As a result, it is unclear how prosocial behavior changes across the life span and whether older adults are sufficiently motivated to perform effortful helping behaviors that may be vital for maintaining social bonds.

Do levels of prosociality change between younger and older adults? *Socioemotional-selectivity theory* suggests that people become more empathic as they age and as a result may become more prosocial (Carstensen, 2006). Moreover, the aging brain undergoes profound neurobiological changes with loss of dopamine transmission (Samanez-Larkin & Knutson, 2015), a neurotransmitter system that has been linked to higher selfishness and lower prosociality (Crockett et al., 2015), of up to 10% per decade. At the population level, older adults donate more money to charity (Charities Aid Foundation, 2012), but lab-based studies using economic games, such as the dictator game, as proxy measures of prosocial behavior have shown both that older adults transfer more money compared with younger adults (Engel, 2011) and no difference between age groups (Rieger & Mata, 2013; Roalf et al., 2011).

However, the designs of such studies may mask real changes in social motivation and conflate potential mechanisms. First, the personal cost in these paradigms is always financial. Yet older adults putatively value economic rewards differently and may have higher accumulated wealth (Mayr & Freund, 2020); further, many everyday prosocial acts do not come at a financial cost for older adults (Cameron et al., 2019; Inzlicht & Hutcherson, 2017). Second, these tasks cannot distinguish changes in self- or other-regarding preferences—more money for the other person equates to less money for oneself. As a result, older adults may or may not show differences in prosocial behavior because they are more motivated to benefit another person, or more trivially, they may simply value their own monetary gains less. Finally, it is plausible that older adults might indulge in virtue signaling and make prosocial choices but be unwilling to incur the real costs required by effortful altruistic acts. By disentangling self- and other-oriented motivation, and by examining costs that are not financial, we can test whether older adults show shifts in levels of prosociality.

One cost that is a crucial factor influencing social behavior is effort. Typically people are averse to exerting effort, and rewards are devalued or discounted by the amount of effort required to obtain them (Chen et al., 2020; Chong et al., 2017; Hartmann et al., 2013; Klein-Flügge et al., 2015; Pessiglione et al., 2018; Shenhav et al., 2017). Many helping acts are also effortful, although this is rarely investigated. Whether it is the physical cost of opening the door for the person behind you or the effort of helping colleagues with their work, these acts require prosocial motivation—a willingness to exert effort to benefit another person.

Importantly, theoretical accounts of effort suggest that there are at least two critical components. First, you must decide whether you are willing to exert effort (Manohar et al., 2015), and second, you have to energize actions appropriately to obtain the desired outcome. A previous study suggested that although young adults chose to help others (prosocial behavior), they were “self-biased” in their motivation, choosing to put in higher levels of physical effort to gain rewards for themselves than for another person (Lockwood, Hamonet, et al., 2017; Mosner et al., 2017). Younger adults also put less energy into prosocial actions than into identical self-benefiting ones. Importantly, several studies have suggested that aging is associated with increased apathy, a reduction in motivation and goal-directed behavior (Van Reekum et al., 2005). Therefore, compared with younger adults, older adults may be even less willing to engage in highly effortful prosocial acts.

**Statement of Relevance**

Social interactions are crucial for maintaining health and well-being, particularly in older adults, for whom social isolation is a major public-health challenge. Social interactions are fundamentally shaped by how willing people are to put in effort to help others. Here, we tested people’s willingness to make effortful helping actions in two groups of adults, one younger and one older. We found that older adults chose to put in more effort to help others than did younger adults. Strikingly, unlike younger adults, older adults also put as much energy into actions to help themselves. These findings suggest that older adults become more prosocially motivated and use relatively more energy when helping others. Therefore, the fundamental nature of human prosociality changes across the life span, which has important implications for theories of prosocial behavior as well as our understanding of healthy aging.

In order to disentangle how motivated older adults are to benefit themselves and others, we tested two groups of adults, one younger and one older, on a physical-effort-based decision-making paradigm (Lockwood, Hamonet, et al., 2017). Participants were given a choice between two options, (a) a higher-effort (30%–70% of their maximum voluntary contraction [MVC] measured on a handheld dynamometer), higher-reward (2–10 credits) work option that varied on each trial and (b) a lower-effort (0% MVC), lower-reward (1 credit) rest option (see Fig. 1). After choosing, participants had to squeeze the dynamometer to the required level of force in order to obtain the credits. If they succeeded, the credits were banked and equated to a bonus payment at the end of the study; if they failed, they got nothing from that trial. Importantly, on half of the trials participants chose between the two options in which they put in the force and received the reward, but on *other* trials, participants made the choice and put in the effort, but the reward was given to the other person. Crucially, this task independently measured effort sensitivity and reward sensitivity, both for self-benefiting and other-benefiting behaviors. Using this design in combination with computational modeling allowed us to examine how much people devalue rewards as a function of the amount of effort required to obtain them (i.e., *by effort*) for themselves and for others in terms of their effort-based decisions and also the degree to which people’s actions were energized.

**Fig. 1.**
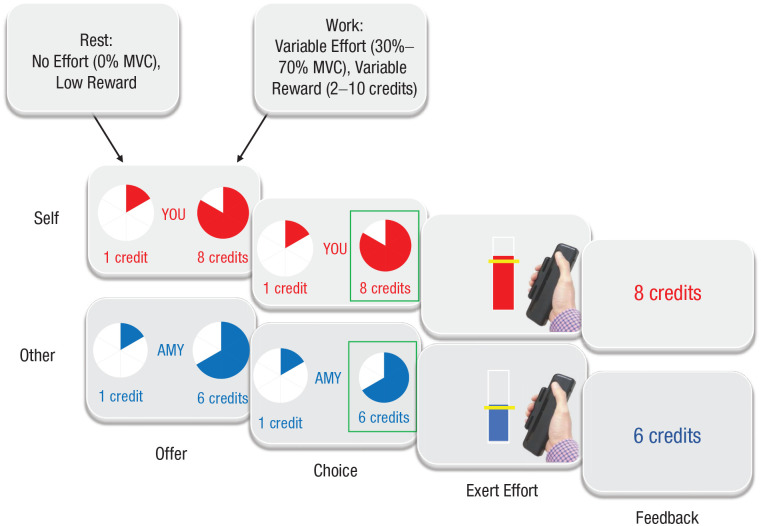
Prosocial-motivation measure. Participants were designated “Player 1” at the beginning of the testing session and told that they would be making decisions that impacted another player who they knew was also in the testing session but they would not meet face to face (see the Method section). Maximum voluntary contraction (MVC) was measured by asking participants to squeeze as strongly as they could on a handheld dynamometer at the beginning of the experiment. On each trial, they were presented with a rest option, which required no effort (0% MVC) for a low reward of 1 credit, and a work option, which required more effort (30%–70% MVC) but also generated more reward (2–10 credits). After making their selection, participants then had to exert the required force to the correct degree to receive the reward. Visual feedback of the amount of force used was displayed on the screen. Participants were informed that they would have to reach the required force level (marked by the yellow line) for at least 1 s out of a 3-s window. Participants then saw the outcome that corresponded to the offer they had chosen, unless they were unsuccessful, in which case “0 credits” was displayed. Crucially, on *self* trials, participants made the choice, exerted the effort, and received the reward themselves, whereas on *other* trials, participants made the choice and exerted the effort, but the other participant received the reward. Participants completed 150 trials, 75 for themselves and 75 for the other person.

## Method

All data and code used to analyze the data and reproduce the figures are available on OSF at https://osf.io/guqrm. The study was not preregistered.

### Participants

Seven participants were excluded from the study because they reported disbelief in the deception. Data were missing for one participant because of technical error, and one participant did not complete the full study and was excluded from analyses. This left a final sample of 187 participants consisting of 95 younger adults (age: range = 18–36 years, *M* = 24; 56 female) and 92 older adults (age: range = 55–84 years, *M* = 69; 43 female). The sample size was based on a previous study in younger adults (Lockwood, Hamonet, et al., 2017) and a power calculation that showed that we had 91% power to detect a medium effect size (*d* = 0.5) with at least 92 participants in each group. We aimed to test approximately equal numbers of older and younger adults. We initially intended our age ranges to be 20 to 35 and 60 to 85, but during testing, we found that three people were under the age of 20 (youngest age = 18) and four were under the age of 60 (youngest age = 55), making the age ranges look wide. However, excluding these individuals did not change any analysis (see Tables S1–S3 in the Supplemental Material available online), and therefore we decided to include them in the final sample.

Participants were recruited through university databases, social media, and the community. All participants provided written informed consent, and the study was approved by a local ethics committee and a National Health Service Research Ethics Committee. Exclusion criteria included previous or current neurological or psychiatric disorder (as reported by the participants), non-normal or non-corrected-to-normal vision, and (for the older sample) scores on Addenbrooke’s Cognitive Examination III (ACE-III) that indicated potential dementia (cutoff score = 82). Participants were paid £10 per hour and were told that they and the other participant would receive an additional bonus payment of up to £5 at the end of the study on the basis of the number of credits that they earned.

### Design

The task structure was the same as that used in Lockwood, Hamonet, et al.’s (2017) study. Participants completed 150 trials—75 decisions for themselves and 75 decisions for the other person. Each trial involved a choice between a baseline option that consisted of gaining 1 credit for no effort or an alternative experimental offer that varied in the level of effort required (30%, 40%, 50%, 60%, or 70% MVC) and level of reward provided (2, 4, 6, 8, or 10 credits; see Fig. 1). Moreover, we specifically designed the study to minimize any potential effects of fatigue interacting with our effects of interest. Participants were required to squeeze for only 1 s out of a 3-s window to obtain the reward (in trials of 10-s duration) and only when they decided to accept the offer. Moreover, many of the work trials were not very demanding (< 50% of MVC), and three breaks were provided in the study. Finally, we counterbalanced trials such that the same number of *self* and *other* trials with equal effort and reward levels were presented in miniblocks of 50 trials so that any potential fatigue effects would equally affect our experimental conditions.

### Apparatus

Stimulus presentation was programmed on a PC using MATLAB (The MathWorks, Natick, MA) and the Psychophysics Toolbox (Version 3.0; Kleiner et al., 2007). Force was recorded using a handheld TSD121B-MRI clench dynamometer (Biopac Systems, Goleta, CA). The PC screen provided participants with real-time visual feedback on the force being exerted.

### Procedure

#### Role-assignment procedure

To ensure that participants believed that their choices and effort exerted resulted in outcomes for another person, we told them that there was a second participant taking part in the study. They did not see the other participant (who was in fact a confederate), in accordance with the procedure described by Lockwood, Hamonet, et al. (2017). Participants were told that selecting a ball from a box would randomly assign them to the role of either Player 1 or Player 2. Player 1 would play the role of the decider, which meant that they would make decisions that affected both themselves and Player 2, whereas Player 2 would be a receiver, which meant that they would make decisions affecting only themselves. Participants were handed a glove and told not to speak so that their identities could not be discerned. A second experimenter arrived in the room with the confederate; the confederate was handed a second glove but remained on the other side of the door at all times, without ever being seen by the participant. Participants were asked to place their hands in front of the door and wave to one another to ensure that it was clear that there was another person there.

The experimenter then tossed a coin to decide who would pick from the box first. Each participant selected a ball and was told which role in the study they were assigned to. This method ensured that participants could not be influenced by the age of the receiver. We also used names for the receiver participant that were gender matched to the decider participant. To ensure that the two groups did not perceive the receiver participant differently, we asked participants to rate the following questions on a scale from 0 to 9: “How similar do you feel to the other participant?” and “How much do you like the other participant?” There were no significant differences between groups in ratings of similarity, *t*(184) = −0.86, *p* = .39, Cohen’s *d* = −0.12, 95% confidence interval (CI) = [−0.41, 0.16], or liking, *t*(184) = −0.40, *p* = .69, Cohen’s *d* = −0.06, 95% CI = [−0.35, 0.23].

#### Task procedure

Participants were asked to grip a handheld dynamometer with as much force as possible to determine their MVC. This ensured that although individuals differ in strength, the effort levels used in the study would be relative to those differences. This measurement was then used as a participant-specific threshold for the levels of effort required to obtain rewards in the main task; it was repeated twice. Despite determining each participant’s individual threshold and therefore controlling for any potential baseline differences in strength across groups, we also tested whether there were any significant differences between groups in the initial force exerted. We found no statistically significant difference between older adults (0.99 V, *SD* = 0.32) and younger adults (1.12 V, *SD* = 0.62) in MVC (Mann-Whitney *U* test = 4,029, *p* = .357, 95% CI for rank biserial correlation = [−0.239, 0.088]). In addition, this measure of MVC was done before participants received any instruction as to the nature of the task, to ensure that they were not influenced to squeeze less than their maximum to be able to collect more rewards in the task.

In the experimental task, participants made decisions between a baseline low-effort option (0% of MVC) that rewarded them with 1 credit and a variable offer in which more credits (2, 4, 6, 8, or 10 credits) were available but that also required more force (30%, 40%, 50%, 60%, or 70% of the MVC, represented by segments in a pie chart). The effort and reward levels were varied independently over trials, and each effort–reward combination was sampled three times for each recipient. There were 150 trials in total: 75 *self* trials in which participants chose between the offer and the baseline for themselves and 75 *other* trials in which they made these decisions for the other person. To obtain the rewards on each trial, participants had to apply a force that exceeded the required level for a total of 1 s out of a 3-s window. Failure to do this resulted in no reward. The offer of 1 credit was used for the baseline condition to ensure that there was a clear incentive for participants to choose the baseline if the value was not considered worth it, rather than choosing the offer and then not exerting any effort at all. If a choice was not selected, 0 credits were given. All trials, regardless of the choice made (or if no response was made), lasted for the same duration. This ensured that choices were not influenced by discounting effects of temporal delay rather than level of effort. Indeed, success rates were very high (98% in younger adults and 97% in older adults), indicating that participants were almost always able to achieve the required amount of force. The fact that failure rates were so low also helped to rule our potential effects of risk aversion, which may interact with effort discounting, as there was a very high probability that participants would receive the rewards from the options they chose.

Prior to the decision-making task, participants experienced each effort level three times across 18 trials. They also learned to associate each level of effort with the elements in the pie chart: They were instructed that if only one element of the pie chart was shown, then 0% force was required and that this was the baseline offer, equivalent to a rest. However, they still had to grip the dynamometer in their hand. During the training session, only 1 credit was on offer; participants were told that this credit would not count toward their payment, and they did not choose whether to opt out of exerting the effort.

#### Questionnaire assessments and demographics

Older adults were screened for dementia using the ACE-III (Hsieh et al., 2013). A brief screening tool, the ACE-III examines five cognitive domains: attention, memory, language, fluency, and visuospatial abilities. The maximum score on the ACE-III is 100 points, and 82 is the cutoff denoting significant cognitive impairment (higher scores indicate greater impairment).

#### Posttask rating

After the study, participants were asked two questions about how positive they felt when receiving rewards for themselves and for the other participant. Participants indicated their rating using a sliding scale ranging from 0 (*not at all*) to 10 (*very positive*). Posttask ratings were administered using the Qualtrics platform (https://www.qualtrics.com/).

## Results

Groups were matched on gender (*p* = .108) and years of education (*p* = .203; mean years of education for younger adults = 15.4, *SD* = 16.5, range = 11–17; mean years of education for older adults = 15.0, *SD* = 2.77, range = 6–20).

### Older adults devalue rewards by effort less than younger adults, particularly when other people will benefit

**Fig. 2.**
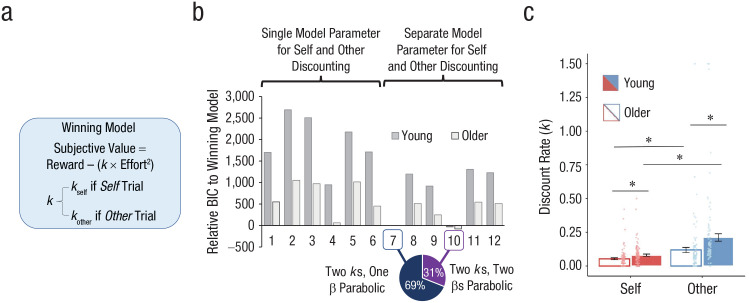
Discount-rate (*k*) modeling. The discount-rate parameters were estimated by a parabolic model (a) that had separate parameters for *self* and *other* trials that had the best fit to participants’ choice behavior. The full model comparison of parabolic, linear, and hyperbolic discounting functions (b) is shown for both single (Models 1–6) and separate (Models 7–12) discount parameters for *self* and *other* trials and single and separate noise (β) parameters for *self* and *other* trials. A parabolic model with separate parameters for *self* and *other* discounting, but a single noise parameter, best explained behavior in the majority of subjects in both groups (Model 7). This model had the lowest summed Bayesian information criterion (BIC) and also explained behavior in the highest percentage of participants. The pie chart shows the percentage of participants for whom the winning model explained behavior (blue), compared with the same model with separate noise parameters (purple). The graph displays the relative BIC of all other models to the BIC of Model 7. Discount-rate parameters from this winning model (c) are shown for the *self* and *other* conditions, separately for young and older participants. Bars show group means, and dots are individual data. Asterisks denote significant differences between conditions (*p* < .01). Error bars show standard errors of the mean.

We fitted a computational model of effort discounting to each participant’s choice behavior to examine the rate at which the two groups discounted rewards by effort. It has previously been shown (Lockwood, Hamonet, et al., 2017) that this model allowed us to parameterize people’s motivation using separate *k* parameters for *self* and *other* trials, plus an additional noise parameter characterizing the stochasticity of choices (β; see Fig. 2a and b). We used this previously validated model to assess whether there were differences in the discounting rate as a function of group (younger and older) and recipient (self and other). The *k* parameter precisely quantifies the rate at which rewards are devalued by effort, with higher *k* parameters indexing steeper discounting, or lower motivation, and lower *k* parameters indicating shallower discounting, or higher motivation.

We analyzed the estimated *k* parameters using robust linear mixed-effects regression, which is robust to the influence of outlier data (using the rlmer function from the *robustlmm* package in R; *robustlmm* Version 2.3; Koller, 2016). With the estimated *k* parameters from the model as the outcome variable, we defined recipient, group, and their interaction as fixed effects and included a subject-level random intercept. This analysis showed a significant Recipient × Group interaction (*b* = −0.039, 95% CI = [−0.067, −0.011], *z* = −2.739, *p* = .006) that was driven by lower discounting in older compared with younger adults, particularly during the *other* condition (Fig. 2c; young vs. old *k* for *other* condition: *z* = 4.90, *p* < .001; young vs. old *k* for *self* condition: *z* = 3.20, *p* = .001). There were also main effects of recipient (*b* = −0.037, 95% CI = [−0.057, −0.017], *z* = −3.656, *p* < .001) and group (*b* = 0.065, 95% CI = [0.045, 0.084], *z* = 6.445, *p* < .001). To account for possible floor effects driving the interaction, we also conducted an additional analysis excluding any *k* values less than 0.01, and all results remained the same (see Table S4 in the Supplemental Material). Therefore, older adults were more prosocial, devaluing rewards by effort less steeply, particularly when the other person would benefit.

### Older and younger adults still distinguish between themselves and others in choices

Could the previous findings be the result of older adults simply not being able to distinguish *self* and *other* trials? We next examined whether older adults differentiated between themselves and others at all by comparing the *self* and *other* discount parameters separately in the two groups. Wilcoxon signed-rank tests showed that both groups distinguished between themselves and others in their choices: Young adults (*z* = −7.74, *p* < .001, 95% CI for rank-biserial correlation = [−.949, −.876]) and older adults (*z* = −6.40, *p* < .001, 95% CI for rank-biserial correlation = [−.849, −.653]) had significantly higher discount parameters for *other* compared with *self* trials. This replicates the findings of Lockwood, Hamonet, et al. (2017) in the younger adults but extends them to older adults, showing that although older adults are more motivated for others than are younger adults, they are still more motivated to benefit themselves than others.

To further support the notion that older adults can still distinguish between themselves and others but show less of a self-bias in their choices, and also to test whether our model had good explanatory power in the current sample, we performed a model comparison (Lockwood & Klein-Flügge, 2020). We compared our chosen model with a range of other possible models that had either separate or singular *k* parameters and β parameters for *self* and *other* trials. We also compared different plausible mathematical functions that could account for discounting behavior in this task—linear, parabolic, and hyperbolic (Chen et al., 2020; Chong et al., 2017; Hartmann et al., 2013; Lockwood, Hamonet, et al., 2017). This resulted in two classes of models, one that had the same *k* to characterize discounting on *self* and *other* trials (Models 1–6) and one class with separate *k*s (Models 7–12; see Fig. 2b and also the Supplemental Material). Within these models, we tested a further two classes of models that characterized whether separate parameters for levels of noise (β, softmax function; Models 4–6 and 10–12) or single parameters for noise (Models 1–3 and 7–9) best explained behavior. Models were fitted to behavioral data using the softmax function (for further model-fitting details, see the Supplemental Material).

As predicted, the winning model in both younger and older adults was the same parabolic model reported previously by Lockwood, Hamonet, et al. (2017) and in the analyses outlined above, in which separate parameters characterized the devaluation of rewards for *self* and *other* trials (Figs. 2a and 2b). This winning model was able to explain behavior (i.e., had the lowest Bayesian information criterion [BIC]) in the majority of participants (69.5% of younger participants, 68.5% of older participants) but was very close in BIC score to an alternative model that also had separate discount parameters but also separate βs, a pattern we also found in our previous study (see the Supplemental Material). We also further validated our winning model in two ways. First, we calculated the median *R*^2^ for the model and found that the model was able to explain 86% (*SD* = 11%) of the variance in choices in older adults and 85% (*SD* = 10%) of the variance in choices in younger adults. We also performed a parameter recovery (Lockwood & Klein-Flügge, 2020; Palminteri et al., 2017) to show that parameters from our best-fitting model were recoverable in simulated data based on our schedule. We showed good recovery of the three parameters (self: *k* = 93%, other: *k* = 93%, β = 77%; for further details, see the Supplemental Material). Together these analyses show that our winning model could accurately describe behavior in both young and older adults.

**Fig. 3.**
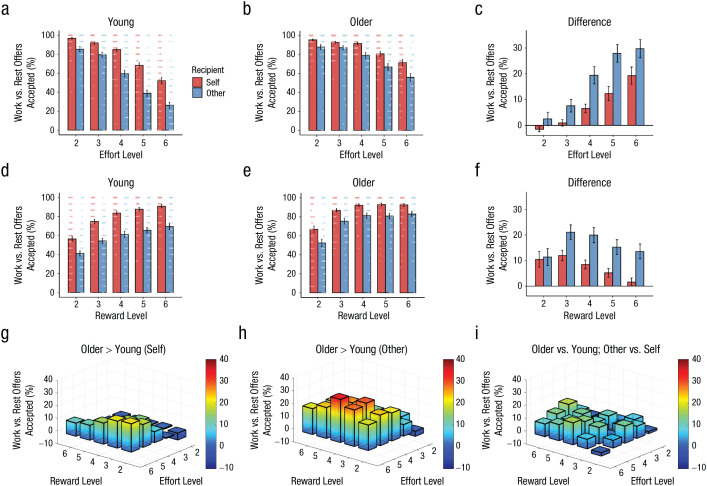
Mean percentage of work versus rest offers accepted for the self and others. The top row shows the percentage of participants who exerted each level of effort for themselves and for others, separately for (a) young adults, (b) older adults, and (c) the difference between the two groups (older – younger). The middle row shows the percentage of participants who exerted effort for each reward level for themselves and for others, separately for (d) young adults, (e) older adults, and (f) the difference between the two groups (older – younger). In the bottom row, the 3-D plots show choices across different effort and reward levels (g) in young compared with older adults in the *self* condition, (h) in young compared with older adults in the *other* condition, and (i) in older adults, compared with younger adults, plotted for choices to help others compared with themselves. In the top and middle rows, dots show individual data, and bars show means. Error bars show standard errors of the mean.

To support these model-based analyses, we analyzed the choice data with a generalized linear mixed-effects model using the glmer function from the *lme4* package in R (*lme4* Version 1.1.26; Bates et al., 2015). Analyses of the choice data in this way also enabled us to test separately for the influences of effort and reward on choices, which were combined in the computational *k*-parameter analysis. With choice coded as a binary outcome variable, we defined group, recipient, effort level, reward level, and their interactions as fixed effects. We included a subject-level random intercept and tested the fixed effects for statistical significance using a Type II Wald χ^2^ test. Mirroring the model-based results, this analysis showed a significant Group × Recipient × Effort × Reward interaction, χ^2^(16) = 27.774, *p* = .034, suggesting differential influences of recipient, effort, and reward between the two groups (Fig. 3i; see also Table S5 in the Supplemental Material). Related to the four-way interaction, we also observed two-way interactions between group and reward, group and effort, and group and recipient (all *p*s < .05; for full statistical details, see Tables S5 and S6 in the Supplemental Material). Notably, the Group × Effort interaction showed that it was at higher levels of effort (Levels 3–6) that the younger and older groups differed (Figs. 3a to 3c; see also Table S6), and the Group × Recipient interaction showed that the older adults chose to put in more effort for others compared with themselves overall (Table S6). Moreover, because we manipulated reward and effort levels independently, we could also rule out that participants perceived the rewards as differentially salient for themselves and others, driving our effects, because we observed no significant Recipient × Group × Reward interaction. Instead it was the interaction of effort level, reward level, and recipient that distinguished the two groups. Finally, there was a significant Group × Recipient interaction for the total number of points won in the *self* and *other* conditions, with older adults winning relatively more points for the other person (349.38, *SD* = 9.42) compared with younger adults (300.02, *SD* = 95.76; Cohen’s *d* = 0.72, 95% CI = [0.42, 1.01]; Group × Recipient, *p* = .003).

Therefore, across model parameters, model comparison, and mixed-model statistical analyses, our results were consistent: Older adults’ prosocial decisions differed from younger adults’ not because of trivial changes in their sensitivity to money or decision noise but because they evaluated rewards and effort differently when making prosocial decisions. In summary, older adults were more motivated to exert higher levels of effort for higher rewards when other people would benefit.

### Older adults show no self-bias when energizing actions

A second crucial aspect of prosocial behavior is to what extent people actually energize the actions required after they decide to help someone. Previous studies have shown that younger adults energize their actions less when another person will benefit than when they themselves will benefit at higher levels of effort (Lockwood, Hamonet, et al., 2017). We found that older adults are more prosocially motivated, but do they also energize their actions to the same degree when someone else is the beneficiary?

**Fig. 4.**
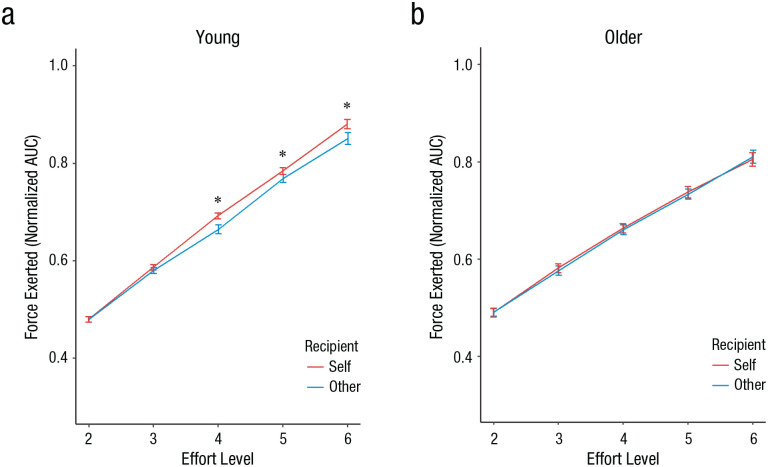
Mean area under the curve (AUC) during the 3-s force period, normalized to the maximum level of force participants exerted across trials, as a function of participants’ level of effort. Results are shown separately for (a) young participants and (b) older participants in both the *self* and *other* conditions. Error bars show standard errors of the mean. Asterisks indicate significant Group × Recipient interactions (all *p*s < .012). For plots displaying all data points, see Figure S1 in the Supplemental Material available online.

To answer this question, we used the lmer function in *lme4* (Bates et al., 2015) to run a linear mixed-effects model to predict the force that participants exerted on each trial. For this analysis, we normalized participants’ force as a proportion of their maximum force to account for between-subjects variability in force exerted; we then calculated the area under the curve for the 3-s window in which they exerted force. Our model predicted normalized force as a continuous variable with a subject-level random intercept. Effort level, reward level, recipient, group, and their interactions were included in the model. Intriguingly, we found a significant three-way interaction among group, effort, and recipient, χ^2^(4) = 25.956, *p* < .001 (Figs. 4a and 4b; see also Tables S7 and S8 in the Supplemental Material). This showed that at higher levels of effort, young adults exerted more force when rewards benefited themselves than others (Group × Recipient interaction was significant at effort Levels 4, 5, and 6, all *p*s < .012; see the text and Tables S7 and S8 in the Supplemental Material). Older adults showed no difference in force exertion between the *self* and *other* conditions, suggesting a loss of the self-bias compared with younger adults. There was also a significant Group × Effort × Reward interaction, χ^2^(16) = 27.579, *p* = .035; two-way interactions for the effects of group and recipient, group and effort, and recipient and effort; and main effects of effort, reward, and recipient (all *p*s < .05; for post hoc comparisons, see Table S8). Importantly, there were no between-group differences in the percentage who were successful once they had chosen to work for themselves and others (young adults’ mean success rate = 0.98, *SD* = 0.03; older adults’ mean success rate = 0.97, *SD* = 0.05; *p* = .107, Cohen’s *d* = 0.24, 95% CI = [−0.04, 0.53]), and there were no significant effects of group, recipient, or their interactions when we ran a model predicting success on each trial—group: χ^2^(1) = 0.519, *p* = .471; recipient: χ^2^(1) = 0.855, *p* = .355; interaction: χ^2^(1) = 1.535, *p* = .215. Finally, we ran an analysis also excluding trials in which participants failed, but all results remained significant (see Table S9 in the Supplemental Material). This suggests that differences in the energization of action between the two groups were not driven by increased failure rates.

### Individual differences in self-reported positivity and decision-making

Socioemotional-selectivity theory posits that as people get older they focus more on their emotional states, such as empathy (Carstensen, 2006). Such an account would predict that individual differences in effort discounting (*k*) might be related to how positive people felt when obtaining rewards for others. Moreover, theoretical accounts of prosocial behavior have suggested that one motivation for prosocial behavior is the warm glow one gets from helping another person (Andreoni, 1990). However, it is unknown whether older adults experienced greater positivity at helping others than did younger adults in our study, and whether this warm glow is maintained across the life span. Finally, we also sought to test age-relevant differences in how positive participants felt about putting in effort to reward themselves and whether such positivity was correlated with their willingness to put in effort for their own benefit. Because we observed a reduced self-bias in older adults, we examined whether there was still an association in this group between feelings of positivity and choosing to help oneself. Therefore, after completing the main task, participants rated the following questions: “How positive did you feel when you won credits for [the other participant/yourself]?’’ (0 = *not at all*, 10 = *very positive*). One participant in the older group did not complete the self-report ratings, leaving a sample of 91 for that group.

**Fig. 5.**
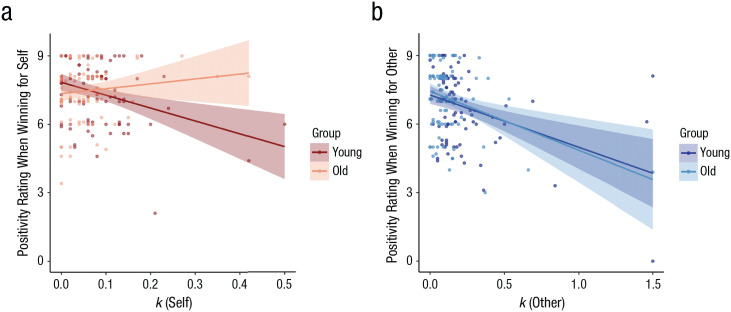
Posttask positivity ratings when winning rewards (a) for oneself and (b) the other person as a function of discounting rate and age group. Participants rated how positive they felt (0 = *not at all*, 10 = *very positive*) when winning credits for themselves and when winning credits for others at the end of the experiment. Dots show individual data, and slopes show best-fitting regressions. Shaded areas around slopes indicate 95% confidence intervals.

In younger adults, the *self* and *other* discounting parameters were both significantly negatively associated with the respective subjective rating. Self-discounting (*k*) was related to subjective positivity in the *self* condition, *r*(93) = −.328, *p* = .001, 95% CI = [−.136, −.497], and *other* discounting related to subjective positivity in the *other* condition, *r*(93) = −.382, *p* = .0001, 95% CI = [−.196, −.542] (Fig. 5). The more positive people felt when getting rewards for themselves or the other person, the more effort they put in (indexed by lower discounting) for themselves and the other person, respectively. However, in older adults, *k* for the *other* condition was significantly correlated with positivity winning credits for other people, *r*(89) = −.326, *p* = .002, 95% CI = [−.128, −.498], but *k* for the *self* condition was not significantly associated with self-rated positivity, *r*(89) = .115, *p* = .277, 95% CI = [−.093, .314]. Importantly, the correlations for self *k* and self positivity were significantly different between groups (*z* = 3.06, *p* = .002, using the paired.r function in the *psych* package; Version 2.0.9; Revelle, 2015). This suggests that feelings of positivity at rewarding other people are related to the balance of effort exerted and reward gained in both younger and older adults. However, although younger adults’ feelings of positivity at rewarding themselves were related to a balance of effort and reward, older adults discounting for themselves was not related to how positive it made them feel.

We next examined whether this difference between groups in positivity ratings and association with *k* was related to changes in how participants felt overall when putting in effort to win rewards for themselves and others. There were no differences between groups in overall mean ratings of positivity in either the *self* or *other* condition, as both younger adults (self: *M* = 7.39, *SD* = 1.36; other: *M* = 6.79, *SD* = 1.61), *t*(94) = 3.29, *p* = .001, *d* = 0.34, 95% CI = [0.13, 0.55], and older adults (self: *M* = 7.46, *SD* = 1.27; other: *M* = 7.13, *SD* = 1.47), *t*(90) = 2.75, *p* = .007, *d* = 0.29, 95% CI = [0.08, 0.50], reported feeling more positive when winning credits for themselves compared with others (there was no significant interaction between groups; *z* = −1.596, *p* = .111).

## Discussion

Many prosocial behaviors require the motivation to exert effort. Here, we showed that older people, compared with younger people, are more prosocially motivated in two crucial aspects of behavior. First, computational modeling and mixed-effects models show that older adults discount rewards by effort less when benefiting others, and thus they are more willing to choose highly effortful prosocial acts. Second, whereas younger adults show a self-bias, pursuing highly effortful actions that benefited themselves more than others, older adults do not. Thus, greater prosociality was demonstrated not only in older adults’ decisions but also in how much energy they allocated to self- and other-benefitting acts. Finally, we observed individual differences in the relationship between discounting in the two groups and their feelings of positivity at helping themselves and others. Positive feelings toward rewarding others were correlated with the willingness to put in effort for others in both younger and older adults, consistent with a maintained sense of “warm glow” across the life span, but only in younger adults did the willingness to put in effort for themselves correlate with how positive the rewards made them feel. Overall, we found, across several indices, that older adults are more prosocial than younger adults and have a lower self-favoring bias in their effort-based decision-making. Therefore, prosocial behavior could fundamentally shift across the life span.

Studies examining life-span changes in prosocial behavior have been mixed. Here, we showed that older adults might be more prosocial in social interactions than younger adults, as suggested by some studies using economic games (Sze et al., 2012). However, our approach was able to show that this effect is not because older adults value money differently per se, as the cost was not money but effort. Moreover, this effort cost was adjusted to each person’s capacity and was manipulated independently from reward in separate *self* and *other* conditions, so we were able to identify changes in sensitivity to a cost between a self-benefiting and a prosocial act. Importantly, both in choice behavior and in the energization of actions, there were significant differences between young and older adults’ sensitivity to the effort cost that differed between the *self* and *other* conditions. These findings highlight the necessity to examine effort and self- and other-oriented motivation independently, in order to understand specific life-span changes in prosocial behaviors. In addition, these results highlight the importance of comparing people’s willingness to put effort into different types of behavior and not treat motivation as a unidimensional construct. Indeed, some studies in the cognitive domain have found that older adults are more averse to effort than younger adults when it comes to cognitive effort (Hess & Ennis, 2012; Westbrook et al., 2013) and also that cognitive and physical efforts are valued differently (Chong et al., 2017). Dissecting the different components of effort-based decision-making in various contexts will be crucial for accurately quantifying and unpacking the mechanisms underlying multiple facets of people’s motivation (Ang et al., 2017; Cameron et al., 2019; Chong et al., 2017; Inzlicht & Hutcherson, 2017; Kool & Botvinick, 2018; Lockwood, Ang, et al., 2017).

Why might older adults be more prosocial when deciding to put in effort and energize their actions? There are several possible explanations both at the biological and sociocultural level. Socioemotional-selectivity theory posits that as people grow older, their time horizon shrinks, leading to changes in motivational goals and shifts in priority driven by changes in emotional needs (Beadle et al., 2013; Carstensen, 2006). Evidence in support of this is provided by the observation that antisocial and aggressive behaviors significantly decrease across the life span. Young adults (16–24 years old) have the highest rates of homicide (Office for National Statistics, 2019), and several studies have suggested that criminal activity increases during adolescence and declines in older adulthood (Liberman, 2008). As levels of antisocial behavior and criminality lessen across the life span, it is plausible that such changes would, in parallel, be associated with increased prosociality. However, we did not find much evidence that changes between age groups are linked to higher emotional reactivity. In both groups, how willing someone was to put in effort for another person was positively correlated with how positive they felt when winning points for the other person, and there were no significant difference in the strength of correlation. This would not be entirely consistent with a socioemotional-selectivity account, which would posit that there is a stronger prioritization of this emotional response in older adults. Intriguingly, these results do show that the warm glow linked to how much a person will help others is maintained across the life span, with the caveat that ratings of positivity might be susceptible to experimenter demand effects.

Such findings, as well as the reduced difference between participants’ motivation for themselves and others in both choices and force exerted, suggest that older adults may have lost an emotionally driven self-bias that could lead to their putting in more effort for others compared with themselves, relative to younger adults. There is considerable evidence that young adults show a self-bias in many aspects of cognition and behavior; they prioritize self-relevant over other-relevant information. This includes effort, as shown here, but also other factors. Young adults show a self-bias when learning which of their actions earn rewards for themselves and which arbitrary stimuli belong to them, and they also demonstrate bias in many forms of memory and attention (Lockwood et al., 2016, 2018). Existing studies of changes in self-bias with increased age have been somewhat mixed. One study found an increased emotional-egocentricity bias in older adults (Riva et al., 2016), as measured by the incongruency of *self* and *other* emotional states. A study that employed an associative-matching task suggested a reduced self-bias in older compared with younger adults (Sui & Humphreys, 2017). Here, by independently manipulating costs and benefits on *self* and *other* trials, we found that when it comes to motivation to exert effort, older adults become less self-biased. Future work should begin to distinguish what aspects of the self-bias increase and which decline.

In this study, we specifically focused on willingness to exert physical effort that benefits others—effort that may relate to everyday real-world prosocial acts. Prosocial acts also include behaviors such as doing charitable work or donating money to charity. However, volunteer work can be affected by the amount of time people have available to sacrifice, and monetary donations depend on wealth; both are key issues in aging research on prosocial behavior (Mayr & Freund, 2020). In our task, one major strength was that putting in effort to give rewards to other people had no impact whatsoever on the participant’s own payment at the end. Nevertheless, in future studies, researchers could try to link prosocial effort to everyday prosocial acts, perhaps through measures such as experience sampling, to translate these findings outside the lab. Moreover, researchers could include a measure of perceived wealth to see whether any differences explain variance in how much participants value the monetary rewards on offer. It would also be intriguing to link willingness to exert effort to measures that may quantify social isolation in older adults, such as their social-network size, to examine whether those adults who choose to put in more effort to help others have larger or smaller social networks than younger adults.

Willingness to be prosocial can be affected by social norms such as reciprocity and acceptance (Gintis et al., 2003). We specifically designed our study to minimize these effects: Participants never met face to face, and they were told that they would leave the building at different times and that their identities would never be revealed. However, it could be that social norms are internalized differently across different ages and cultures. It would be interesting for researchers to try to manipulate different social norms in future studies to examine the effect on prosocial choice and force exerted. A strength of the task is that both people’s explicit choices and their implicit energization of action can be measured to provide complimentary insights into prosocial motivation. It would also be important for researchers to examine whether the nature of the receiver changes people’s prosociality, depending perhaps on their age, their closeness, or whether they are perceived as part of an in-group or an out-group. Researchers could also examine whether possible increases in empathy between age groups are linked to differences in willingness to help others: Previous research has suggested that older adults have greater empathic concern for people in need compared with younger adults, although they do not show a benefit from imagining helping others in the same way as younger adults (Sawczak et al., 2019). That also dovetails with research showing an important link between empathy and motivation (Cameron et al., 2019; Lockwood, Ang, et al., 2017). Finally, we note that our results are from a single, albeit well-powered, study, and researchers should seek to replicate our effects in future work.

Overall, we showed that older adults are more prosocial than younger adults in two core components of motivation. Moreover, different emotional considerations may drive decisions in younger and older adults to invest effort to help themselves and others. Understanding the trajectory of social behavior across the life span can inform theoretical accounts of the nature of human prosociality as well as theories of healthy aging—and ultimately, in the long term, help to develop strategies for scaffolding lifelong health and well-being.

## Supplemental Material

sj-docx-1-pss-10.1177_0956797620975781 – Supplemental material for Aging Increases Prosocial Motivation for Effort Supplemental material, sj-docx-1-pss-10.1177_0956797620975781 for Aging Increases Prosocial Motivation for Effort by Patricia L. Lockwood, Ayat Abdurahman, Anthony S. Gabay, Daniel Drew, Marin Tamm, Masud Husain and Matthew A. J. Apps in Psychological Science

## Transparency

*Action Editor:* Patricia J. Bauer

*Editor:* Patricia J. Bauer

*Author Contributions*

M. Husain and M. A. J. Apps contributed equally to this article. P. L. Lockwood, M. A. J. Apps, and M. Husain designed the study. P. L. Lockwood, A. Abdurahman, D. Drew, and M. Tamm collected data. P. L. Lockwood, M. A. J. Apps, A. Abdurahman, and A. S. Gabay analyzed the data. P. L. Lockwood, A. Abdurahman, A. S. Gabay, M. Husain, and M. A. J. Apps wrote the article, and all the authors approved the final version for submission.

**Open Practices:** All data and code used to analyze the data and reproduce the figures have been made publicly available on OSF at https://osf.io/guqrm. The design and analysis plans for the study were not preregistered. This article has received the badge for Open Data. More information about the Open Practices badges can be found at http://www.psychologicalscience.org/publications/badges.

## References

[bibr1-0956797620975781] Andreoni J. (1990). Impure altruism and donations to public goods: A theory of warm-glow giving. The Economic Journal, 100(401), 464–477. 10.2307/2234133

[bibr2-0956797620975781] Ang Y.-S. Lockwood P. Apps M. A. J. Muhammed K. Husain M. (2017). Distinct subtypes of apathy revealed by the apathy motivation index. PLOS ONE, 12(1), Article e0169938. 10.1371/journal.pone.0169938 PMC5226790 28076387

[bibr3-0956797620975781] Bates D. Mächler M. Bolker B. Walker S. (2015). Fitting linear mixed-effects models using lme4. Journal of Statistical Software, 1(1). 10.18637/jss.v067.i01

[bibr4-0956797620975781] Beadle J. N. Sheehan A. H. Dahlben B. Gutchess A. H. (2013). Aging, empathy, and prosociality. The Journals of Gerontology B: Psychological Sciences and Social Sciences, 70(2), 213–222. 10.1093/geronb/gbt091 PMC4415076 24115776

[bibr5-0956797620975781] Cameron C. D. Hutcherson C. A. Ferguson A. M. Scheffer J. A. Hadjiandreou E. Inzlicht M. (2019). Empathy is hard work: People choose to avoid empathy because of its cognitive costs. Journal of Experimental Psychology: General, 148(6), 962–976. 10.1037/xge0000595 30998038

[bibr6-0956797620975781] Carstensen L. L. (2006). The influence of a sense of time on human development. Science, 312(5782), 1913–1915. 10.1126/science.1127488 16809530 PMC2790864

[bibr7-0956797620975781] Charities Aid Foundation. (2012). UK giving 2012. https://www.cafonline.org/about-us/publications/2012-publications/uk-giving-2012

[bibr8-0956797620975781] Chen X. Voets S. Jenkinson N. Galea J. M. (2020). Dopamine-dependent loss aversion during effort-based decision-making. The Journal of Neuroscience, 40, 661–670. 10.1523/JNEUROSCI.1760-19.2019 31727795 PMC6961986

[bibr9-0956797620975781] Chong T. T.-J. Apps M. Giehl K. Sillence A. Grima L. L. Husain M. (2017). Neurocomputational mechanisms underlying subjective valuation of effort costs. PLOS Biology, 15(2), Article e1002598. 10.1371/journal.pbio.1002598 PMC5325181 28234892

[bibr10-0956797620975781] Crockett M. J. Siegel J. Z. Kurth-Nelson Z. Ousdal O. T. Story G. Frieband C. Grosse-Rueskamp J. M. Dayan P. Dolan R. J. (2015). Dissociable effects of serotonin and dopamine on the valuation of harm in moral decision making. Current Biology, 25(14), 1852–1859. 10.1016/j.cub.2015.05.021 26144968 PMC4518463

[bibr11-0956797620975781] Eisenberg N. Cumberland A. Guthrie I. K. Murphy B. C. Shepard S. A. (2005). Age changes in prosocial responding and moral reasoning in adolescence and early adulthood. Journal of Research on Adolescence, 15(3), 235–260. 10.1111/j.1532-7795.2005.00095.x 20592955 PMC2893741

[bibr12-0956797620975781] Engel C. (2011). Dictator games: A meta study. Experimental Economics, 14(4), 583–610.

[bibr13-0956797620975781] Fehr E. Fischbacher U. (2003). The nature of human altruism. Nature, 425(6960), 785–791. 10.1038/nature02043 14574401

[bibr14-0956797620975781] Fratiglioni L. Paillard-Borg S. Winblad B. (2004). An active and socially integrated lifestyle in late life might protect against dementia. The Lancet Neurology, 3(6), 343–353. 10.1016/S1474-4422(04)00767-7 15157849

[bibr15-0956797620975781] Gintis H. Bowles S. Boyd R. Fehr E. (2003). Explaining altruistic behavior in humans. Evolution and Human Behavior, 24(3), 153–172.

[bibr16-0956797620975781] Hartmann M. N. Hager O. M. Tobler P. N. Kaiser S. (2013). Parabolic discounting of monetary rewards by physical effort. Behavioural Processes, 100, 192–196. 10.1016/j.beproc.2013.09.014 24140077

[bibr17-0956797620975781] Hess T. M. Ennis G. E. (2012). Age differences in the effort and costs associated with cognitive activity. The Journals of Gerontology B: Psychological Sciences and Social Sciences, 67(4), 447–455. 10.1093/geronb/gbr129 22131365 PMC3391072

[bibr18-0956797620975781] Hsieh S. Schubert S. Hoon C. Mioshi E. Hodges J. R. (2013). Validation of the Addenbrooke’s Cognitive Examination III in frontotemporal dementia and Alzheimer’s disease. Dementia and Geriatric Cognitive Disorders, 36(3–4), 242–250. 10.1159/000351671 23949210

[bibr19-0956797620975781] Imuta K. Henry J. D. Slaughter V. Selcuk B. Ruffman T. (2016). Theory of mind and prosocial behavior in childhood: A meta-analytic review. Developmental Psychology, 52(8), 1192–1205. 10.1037/dev0000140 27337508

[bibr20-0956797620975781] Inzlicht M. Hutcherson C. A. (2017). Psychology: People work less hard for others. Nature Human Behaviour, 1(7), Article 0148. 10.1038/s41562-017-0148

[bibr21-0956797620975781] Kleiner M. Brainard D. H. Pelli D. G. (2007). What’s new in Psychtoolbox-3? Perception, 36(ECVP Abstract Suppl.).

[bibr22-0956797620975781] Klein-Flügge M. C. Kennerley S. W. Saraiva A. C. Penny W. D. Bestmann S. (2015). Behavioral modeling of human choices reveals dissociable effects of physical effort and temporal delay on reward devaluation. PLOS Computational Biology, 11(3), Article e1004116. 10.1371/journal.pcbi.1004116 PMC4376637 25816114

[bibr23-0956797620975781] Koller M. (2016). robustlmm: An R package for robust estimation of linear mixed-effects models. Journal of Statistical Software, 1(6). 10.18637/jss.v075.i06 PMC7351245 32655332

[bibr24-0956797620975781] Kool W. Botvinick M. (2018). Mental labour. Nature Human Behaviour, 2(12), 899–908. 10.1038/s41562-018-0401-9 30988433

[bibr25-0956797620975781] Liberman A. (2008). The long view of crime: A synthesis of longitudinal research. Springer.

[bibr26-0956797620975781] Lockwood P. L. Ang Y.-S. Husain M. Crockett M. J. (2017). Individual differences in empathy are associated with apathy-motivation. Scientific Reports, 7(1), Article 17293. 10.1038/s41598-017-17415-w PMC5725487 29229968

[bibr27-0956797620975781] Lockwood P. L. Apps M. A. J. Valton V. Viding E. Roiser J. P. (2016). Neurocomputational mechanisms of prosocial learning and links to empathy. Proceedings of the National Academy of Sciences, USA, 113(35), 9763–9768. 10.1073/pnas.1603198113 PMC5024617 27528669

[bibr28-0956797620975781] Lockwood P. L. Hamonet M. Zhang S. H. Ratnavel A. Salmony F. U. Husain M. Apps M. A. J. (2017). Prosocial apathy for helping others when effort is required. Nature Human Behaviour, 1, Article 0131. 10.1038/s41562-017-0131 PMC5555390 28819649

[bibr29-0956797620975781] Lockwood P. L. Klein-Flügge M. C. (2020). Computational modelling of social cognition and behaviour—A reinforcement learning primer. Social Cognitive and Affective Neuroscience. Advance online publication. 10.1093/scan/nsaa040 PMC8343561 32232358

[bibr30-0956797620975781] Lockwood P. L. Wittmann M. K. Apps M. A. J. Klein-Flügge M. C. Crockett M. J. Humphreys G. W. Rushworth M. F. S. (2018). Neural mechanisms for learning self and other ownership. Nature Communications, 9(1), Article 4747. 10.1038/s41467-018-07231-9 PMC6232114 30420714

[bibr31-0956797620975781] Manohar S. G. Chong T. T.-J. Apps M. A. J. Batla A. Stamelou M. Jarman P. R. Bhatia K. P. Husain M. (2015). Reward pays the cost of noise reduction in motor and cognitive control. Current Biology, 25(13), 1707–1716. 10.1016/j.cub.2015.05.038 26096975 PMC4557747

[bibr32-0956797620975781] Mayr U. Freund A. (2020). Do we become more prosocial as we age, and if so, why? Current Directions in Psychological Science, 29(3), 248–254. 10.1177/0963721420910811

[bibr33-0956797620975781] Mosner M. G. Kinard J. L. McWeeny S. Shah J. S. Markiewitz N. D. Damiano-Goodwin C. R. Burchinal M. R. Rutherford H. J. V. Greene R. K. Treadway M. T. Dichter G. S. (2017). Vicarious effort-based decision-making in autism spectrum disorders. Journal of Autism and Developmental Disorders, 47(10), 2992–3006. 10.1007/s10803-017-3220-3 28699053 PMC5711588

[bibr34-0956797620975781] Office for National Statistics. (2019). What do we know about suspects? In Homicide in England and Wales: Year ending March 2018. https://www.ons.gov.uk/peoplepopulationandcommunity/crimeandjustice/articles/homicideinenglandandwales/yearendingmarch2018#what-do-we-know-about-suspects

[bibr35-0956797620975781] Palminteri S. Wyart V. Koechlin E. (2017). The importance of falsification in computational cognitive modeling. Trends in Cognitive Sciences, 21(6), 425–433. 10.1016/j.tics.2017.03.011 28476348

[bibr36-0956797620975781] Pessiglione M. Vinckier F. Bouret S. Daunizeau J. Le Bouc R. (2018). Why not try harder? Computational approach to motivation deficits in neuro-psychiatric diseases. Brain: A Journal of Neurology, 141(3), 629–650. 10.1093/brain/awx278 29194534

[bibr37-0956797620975781] Revelle W. (2015). psych: Procedures for psychological, psychometric, and personality research (Version 2.0.9) [Computer software]. https://cran.r-project.org/web/packages/psych/index.html

[bibr38-0956797620975781] Rieger M. Mata R. (2013). On the generality of age differences in social and nonsocial decision making. The Journals of Gerontology B: Psychological Sciences and Social Sciences, 70(2), 200–212. 10.1093/geronb/gbt088 24056582

[bibr39-0956797620975781] Riva F. Triscoli C. Lamm C. Carnaghi A. Silani G. (2016). Emotional egocentricity bias across the life-span. Frontiers in Aging Neuroscience, 8, Article 00074. 10.3389/fnagi.2016.00074 PMC4844617 27199731

[bibr40-0956797620975781] Roalf D. R. Mitchell S. H. Harbaugh W. T. Janowsky J. S. (2011). Risk, reward, and economic decision making in aging. The Journals of Gerontology B: Psychological Sciences and Social Sciences, 67(3), 289–298. 21926401 10.1093/geronb/gbr099 PMC3325085

[bibr41-0956797620975781] Samanez-Larkin G. R. Knutson B. (2015). Decision making in the ageing brain: Changes in affective and motivational circuits. Nature Reviews Neuroscience, 16(5), 278–289. 10.1038/nrn3917 25873038 PMC5645075

[bibr42-0956797620975781] Sawczak C. McAndrews M. P. Gaesser B. Moscovitch M. (2019). Episodic simulation and empathy in older adults and patients with unilateral medial temporal lobe excisions. Neuropsychologia, 135, Article 107243. 10.1016/j.neuropsychologia.2019.107243 31698010

[bibr43-0956797620975781] Shenhav A. Musslick S. Lieder F. Kool W. Griffiths T. L. Cohen J. D. Botvinick M. M. (2017). Toward a rational and mechanistic account of mental effort. Annual Review of Neuroscience, 40, 99–124. 10.1146/annurev-neuro-072116-031526 28375769

[bibr44-0956797620975781] Sui J. Humphreys G. W. (2017). Aging enhances cognitive biases to friends but not the self. Psychonomic Bulletin & Review, 24(6), 2021–2030. 10.3758/s13423-017-1264-1 28315168

[bibr45-0956797620975781] Sze J. A. Gyurak A. Goodkind M. S. Levenson R. W. (2012). Greater emotional empathy and prosocial behavior in late life. Emotion, 12(5), 1129–1140. 10.1037/a0025011 21859198 PMC3766763

[bibr46-0956797620975781] Van Reekum R. Stuss D. T. Ostrander L. (2005). Apathy: Why care? Journal of Neuropsychiatry and Clinical Neurosciences, 17(1), 7–19. 10.1176/appi.neuropsych.17.1.7 15746478

[bibr47-0956797620975781] Westbrook A. Kester D. Braver T. S. (2013). What is the subjective cost of cognitive effort? Load, trait, and aging effects revealed by economic preference. PLOS ONE, 8(7), Article e68210. 10.1371/journal.pone.0068210 PMC3718823 23894295

