# Supplementary material for: Aging Increases Prosocial Motivation for Effort
Source: Psychol Sci. 2021 Apr 16;32(5):668–81. doi: 10.1177/0956797620975781 (PMC7611497; doi:10.1177/0956797620975781)
Supplement: sj-docx-1-pss-10.1177_0956797620975781 – Supplemental material for Aging Increases Prosocial Motivation for Effort [file sj-docx-1-pss-10.1177_0956797620975781.docx]

“Aging Increases Prosocial Motivation for Effort”

**Supplementary Information Text**

**Model selection and comparison**

To precisely quantify the *subjective* influence of effort on rewards for self and other we created a range of computational models that characterised how rewards were being devalued by the amount of effort. This approach allowed us to quantify people’s motivation to put in effort to reward others using a single parameter, that meaningfully characterizes how motivation is influenced by the balance between effort and reward. Each model contained idiosyncratic  parameters characterizing (‘*K*’) the degree to which a reward was devalued by effort, and ‘noise’ parameters characterizing the stochasticity of choices (β). There were two features that were varied to create the model space. First, we varied the mathematical function that characterises the form of the discounting (i.e., whether rewards are devalued linearly, hyperbolically, or parabolically by physical effort (Chen et al., 2019; Chong et al., 2017; Hartmann et al., 2013; Klein-Flugge et al., 2015; Lockwood et al., 2017)). Second, we compared models which tested whether people devalue or ‘discount’ rewards by effort to the same degree on self or other trials, or instead used separate discounting rates. We therefore created two classes of models that had either the same parameters to characterise discounting (*K*) on self and other trials (models 1-6) or separate ones (models 7-12, **Fig 1.**). Within these models, we tested a further two classes of models that characterized whether separate parameters for levels of noise (β, softmax) (models 4-6, 10-12), or single parameters for noise (models 1-3, 7-9) best explained behaviour. Models were fitted to behavioural data using the fmincon function in Matlab. For all model fitting code, see [https://osf.io/guqrm].

As predicted, the winning model in both younger and older adults was the same parabolic model as reported previously, in line with many previous studies (Chen et al., 2019; Chong et al., 2017; Hartmann et al., 2013; Klein-Flugge et al., 2015; Lockwood et al., 2017), and in the current analyses in which separate parameters characterised the devaluation of rewards for self and other trials, but with a single noise parameter (BIC two discount, one noise parameter younger(BIC) = 10084.83; older(BIC) = 8852.13) (**Figure 2a,b**). Importantly, this model was able to explain behaviour in the majority of participants (younger 69.5% of participants, older 68.5% of participants), but was very close in BIC score to an alternative model that also had separate discount parameters but also separate betas (model 10, younger(BIC) = 10054.83; older(BIC) = 8773.47). Note that this was also the case in our previous study where both models were very close in terms of model fit but the model that won in the majority of participants in two independent samples was model 7, that contained a single noise parameter. Specifically, in this model SV is the subjective net-value of a variable offer of given Effort (E) and Reward (R). The extent to which rewards are subjectively discounted is dependent on the discount parameter (K) which is different on self and other trials:

$$SV=R-KE^{2}$$

$$K=\left\{ \begin{aligned} Kself if self trial \\ Kother if other trial \end{aligned} \right.$$

A high K indicates participants are discounting rewards by the effort to a higher degree. Discount parameters (k’s) were bounded between 0 and 1.5 to ensure parameters reflected a sensible range of behaviour in the task.

Our winning model contained 3 parameters (kSelf, kOther, 𝛽). To assess whether the model accurately predicted behaviour in addition to performing a model comparison we performed a parameter recovery analysis as recommended in the field for studies using a ‘data first’ approach (Palminteri et al., 2017). We simulated behaviour using the same schedule given to our participants. We used a wide range of parameter values (total of 5120 combinations), from a grid of values in the ranges: kSelf=[0.05:0.1:1.5], kOther=[0.05:0.1:1.5], 𝛽=[0.00:0.5:10]. We added noise to each of the five parameters for each simulated agent (from a standard normal distribution multiplied by 0.05) to improve our coverage of possible parameter values. After having generated the behaviour, we refitted the simulated behaviour using fmincon in Matlab. The correlations (r values) between the true simulated and fitted parameter values were: kSelf = .96, Kother = .96, Beta = .77. Thus, parameter recovery was reliable for all parameters.

**Table S1: Analysis of Deviance (Type II Wald test) on choice data GLMM, excluding participants outside of defined age-range**

|  | **Chi-squared** | **Df** | **p-value** |
| --- | --- | --- | --- |
| Group | 16.477 | 1 | < 0.001 |
| Recipient | 1340.147 | 1 | < 0.001 |
| Effort | 3240.344 | 4 | < 0.001 |
| Reward | 2003.934 | 4 | < 0.001 |
| Group:Recipient | 38.301 | 1 | < 0.001 |
| Group:Effort | 140.405 | 4 | < 0.001 |
| Recipient:Effort | 12.379 | 4 | 0.015 |
| Group:Reward | 26.841 | 4 | < 0.001 |
| Recipient:Reward | 56.7 | 4 | < 0.001 |
| Effort:Reward | 95.753 | 16 | < 0.001 |
| Group:Recipient:Effort | 0.862 | 4 | 0.930 |
| Group:Recipient:Reward | 2.426 | 4 | 0.658 |
| Group:Effort:Reward | 22.600 | 16 | 0.125 |
| Recipient:Effort:Reward | 31.561 | 16 | 0.011 |
| Group:Recipient:Effort:Reward | 28.632 | 16 | 0.027 |

**Generalised linear mixed effect model on choice data.** With the choice coded as a binary outcome variable, we defined group, recipient, effort level, reward level, and their interactions as fixed effects. We included a subject-level random intercept.

**Table S2: Analysis of Deviance (Type II Wald test) on force data LMM, excluding participants outside of defined age-range**

|  | **Chi-squared** | **Df** | ***p*-value** |
| --- | --- | --- | --- |
| Group | 2.693 | 1 | 0.101 |
| Effort | 44065.197 | 4 | < 0.001 |
| Reward | 192.716 | 4 | < 0.001 |
| Recipient | 42.136 | 1 | < 0.001 |
| Group:Effort | 428.476 | 4 | < 0.001 |
| Group:Reward | 3.981 | 4 | 0.409 |
| Effort:Reward | 113.013 | 16 | < 0.001 |
| Group:Recipient | 12.358 | 1 | < 0.001 |
| Effort:Recipient | 27.071 | 4 | < 0.001 |
| Reward:Recipient | 20.399 | 4 | < 0.001 |
| Group:Effort:Reward | 27.593 | 16 | 0.035 |
| Group:Effort:Recipient | 23.776 | 4 | < 0.001 |
| Group:Reward:Recipient | 3.049 | 4 | 0.550 |
| Effort:Reward:Recipient | 49.045 | 16 | < 0.001 |
| Group:Effort:Reward:Recipient | 16.939 | 16 | 0.390 |

**Linear mixed effects model on force data.** With the force coded as a continuous outcome variable, we defined group, recipient, effort level, reward level, and their interactions as fixed effects. We included a subject-level random intercept.

**Table S3: Model output for robust linear mixed effects model on k parameters, excluding participants outside of defined age-range**

|  | **Beta** | **SE** | ***z*** | ***p*-value** |  |
| --- | --- | --- | --- | --- | --- |
| Group | 0.065 | 0.010 | 6.445 | < 0.001 |  |
| Recipient | -0.037 | 0.010 | -3.656 | < 0.001 |  |
| Group:Recipient | -0.039 | 0.014 | -2.739 | 0.006 |  |

**Table S4: Model output for robust linear mixed effects model on k parameters, excluding those values < 0.01 to account for floor effects**

|  | **Beta** | **SE** | ***z*** | ***p*-value** |  |
| --- | --- | --- | --- | --- | --- |
| Group | 0.052 | 0.009 | 5.533 | < 0.001 |  |
| Recipient | -0.034 | 0.007 | -5.246 | < 0.001 |  |
| Group:Recipient | -0.023 | 0.009 | -2.581 | 0.01 |  |

**Table S5: Analysis of Deviance (Type II Wald test) on choice data GLMM**

|  | **Chi-squared** | **Df** | **p-value** |
| --- | --- | --- | --- |
| Group | 19.942 | 1 | < 0.001 |
| Recipient | 1393.553 | 1 | < 0.001 |
| Effort | 3345.030 | 4 | < 0.001 |
| Reward | 2076.336 | 4 | < 0.001 |
| Group:Recipient | 42.079 | 1 | < 0.001 |
| Group:Effort | 142.930 | 4 | < 0.001 |
| Recipient:Effort | 14.060 | 4 | 0.007 |
| Group:Reward | 30.516 | 4 | < 0.001 |
| Recipient:Reward | 60.165 | 4 | < 0.001 |
| Effort:Reward | 91.685 | 16 | < 0.001 |
| Group:Recipient:Effort | 0.566 | 4 | 0.966 |
| Group:Recipient:Reward | 3.528 | 4 | 0.473 |
| Group:Effort:Reward | 20.823 | 16 | 0.185 |
| Recipient:Effort:Reward | 33.088 | 16 | 0.007 |
| Group:Recipient:Effort:Reward | 27.774 | 16 | 0.034 |

**Generalised linear mixed effect model on choice data.** With the choice coded as a binary outcome variable, we defined group, recipient, effort level, reward level, and their interactions as fixed effects. We included a subject-level random intercept.

**Table S6: Post-hoc comparisons on the choice data**

| **Interaction** | **Comparison** | **Odds Ratio** | **95% CIs** | | **Bonferroni-corrected**  ***p-*value** |
| --- | --- | --- | --- | --- | --- |
|  |  |  | **Lower** | **Upper** |  |
| **Group-by-Recipient** | Older self vs. Young self | 0.51 | 0.28 | 0.91 | 0.046 |
|  | Older other vs Young other | 0.27 | 0.16 | 0.48 | < 0.001 |
| **Group-by-effort** | Young vs Older, effort level 2 | 1.11 | 0.59 | 2.11 | 1.00 |
|  | Young vs Older, effort level 3 | 0.44 | 0.24 | 0.82 | 0.047 |
|  | Young vs Older, effort level 4 | 0.31 | 0.17 | 0.57 | 0.001 |
|  | Young vs Older, effort level 5 | 0.23 | 0.13 | 0.41 | < 0.001 |
|  | Young vs Older, effort level 6 | 0.20 | 0.11 | 0.36 | < 0.001 |
| **Recipient-by-effort** | Self vs Other, effort level 2 | 0.14 | 0.10 | 0.19 | < 0.001 |
|  | Self vs Other, effort level 3 | 0.23 | 0.17 | 0.29 | < 0.001 |
|  | Self vs Other, effort level 4 | 0.15 | 0.12 | 0.18 | < 0.001 |
|  | Self vs Other, effort level 5 | 0.20 | 0.17 | 0.23 | < 0.001 |
|  | Self vs Other, effort level 6 | 0.23 | 0.19 | 0.26 | < 0.001 |
| **Group-by-reward** | Young vs Older, reward level 2 | 2.68 | 1.51 | 4.75 | 0.004 |
|  | Young vs Older, reward level 3 | 3.89 | 2.16 | 7.01 | < 0.001 |
|  | Young vs Older, reward level 4 | 3.50 | 1.92 | 6.37 | < 0.001 |
|  | Young vs Older, reward level 5 | 2.45 | 1.33 | 4.51 | 0.020 |
|  | Young vs Older, reward level 6 | 1.55 | 0.84 | 2.87 | 0.815 |
| **Recipient-by-reward** | Self vs Other, reward level 2 | 0.33 | 0.28 | 0.38 | < 0.001 |
|  | Self vs Other, reward level 3 | 0.21 | 0.17 | 0.25 | < 0.001 |
|  | Self vs Other, reward level 4 | 0.17 | 0.14 | 0.21 |  |
|  | Self vs Other, reward level 5 | 0.14 | 0.11 | 0.18 | < 0.001 |
|  | Self vs Other, reward level 6 | 0.13 | 0.10 | 0.17 | < 0.001 |

**Post-hoc comparisons for choice data.** We used the *emmeans* package in R to extract the marginal means and perform post-hoc analyses on the choice data.

**Table S7:Analysis of Deviance (Type II Wald test) on force data LMM**

|  | **Chi-squared** | **Df** | ***p*-value** |
| --- | --- | --- | --- |
| Group | 3.056 | 1 | 0.080 |
| Effort | 46894.612 | 4 | < 0.001 |
| Reward | 189.133 | 4 | < 0.001 |
| Recipient | 42.790 | 1 | < 0.001 |
| Group:Effort | 471.504 | 4 | < 0.001 |
| Group:Reward | 4.059 | 4 | 0.398 |
| Effort:Reward | 113.137 | 16 | < 0.001 |
| Group:Recipient | 13.342 | 1 | < 0.001 |
| Effort:Recipient | 24.833 | 4 | < 0.001 |
| Reward:Recipient | 20.641 | 4 | < 0.001 |
| Group:Effort:Reward | 27.579 | 16 | 0.035 |
| Group:Effort:Recipient | 25.956 | 4 | < 0.001 |
| Group:Reward:Recipient | 3.789 | 4 | 0.435 |
| Effort:Reward:Recipient | 46.365 | 16 | < 0.001 |
| Group:Effort:Reward:Recipient | 15.874 | 16 | 0.462 |

**Linear mixed effects model on force data.** With the force coded as a continuous outcome variable, we defined group, recipient, effort level, reward level, and their interactions as fixed effects. We included a subject-level random intercept.

**Table S8:Post-hoc comparisons on the force data**

| **Interaction** | **Comparison** | **Estimated difference** | **95% CIs** | | **Bonferroni-corrected**  ***p-*value** |
| --- | --- | --- | --- | --- | --- |
|  |  |  | **Lower** | **Upper** |  |
| **Group-by-Recipient** | Older self vs. Young self | 0.03 | 0.01 | 0.05 | 0.009 |
|  | Older other vs Young other | 0.02 | 0.00 | 0.04 | 0.161 |
| **Group-by-effort** | Young vs Older, effort level 2 | -0.01 | -0.09 | 0.01 | 1.00 |
|  | Young vs Older, effort level 3 | 0.01 | -0.02 | 0.03 | 1.00 |
|  | Young vs Older, effort level 4 | 0.02 | 0.00 | 0.04 | 0.263 |
|  | Young vs Older, effort level 5 | 0.04 | 0.02 | 0.06 | 0.002 |
|  | Young vs Older, effort level 6 | 0.06 | 0.04 | 0.09 | < 0.001 |
| **Recipient-by-effort** | Self vs Other, effort level 2 | 0.00 | -0.004 | 0.003 | 1.00 |
|  | Self vs Other, effort level 3 | -0.006 | 0.010 | 0.002 | 0.039 |
|  | Self vs Other, effort level 4 | -0.013 | -0.018 | -0.009 | < 0.001 |
|  | Self vs Other, effort level 5 | -0.007 | -0.013 | -0.001 | 0.074 |
|  | Self vs Other, effort level 6 | -0.020 | -0.27 | -0.013 | < 0.001 |
| **Recipient-by-reward** | Self vs Other, reward level 2 | -0.009 | -0.016 | -0.002 | 0.041 |
|  | Self vs Other, reward level 3 | -0.011 | -0.016 | -0.006 | 0.001 |
|  | Self vs Other, reward level 4 | -0.011 | -0.19 | -0.009 | < 0.001 |
|  | Self vs Other, reward level 5 | -0.012 | -0.017 | -0.008 | < 0.001 |
|  | Self vs Other, reward level 6 | 0.00 | -0.005 | 0.004 | 1.00 |
| **Group-by-Recipient-by-Effort** | Group-by-Recipient interaction at effort level 2 | -0.002 | -0.011 | 0.001 | 0.519 |
|  | Group-by-Recipient interaction at effort level 3 | 0.001 | -0.001 | 0.009 | 0.904 |
|  | Group-by-Recipient interaction at effort level 4 | 0.016 | 0.007 | 0.026 | 0.001 |
|  | Group-by-Recipient interaction at effort level 5 | 0.015 | 0.003 | 0.026 | 0.012 |
|  | Group-by-Recipient interaction at effort level 6 | 0.029 | 0.016 | 0.042 | < 0.001 |

**Table S9: Analysis of Deviance (Type II Wald test) on force data LMM, excluding failed trials**

|  | **Chi-squared** | **Df** | ***p*-value** |
| --- | --- | --- | --- |
| Group | 2.486 | 1 | 0.114 |
| Effort | 66168.026 | 4 | < 0.001 |
| Reward | 169.789 | 4 | < 0.001 |
| Recipient | 41.404 | 1 | < 0.001 |
| Group:Effort | 492.489 | 4 | < 0.001 |
| Group:Reward | 4.059 | 4 | < 0.001 |
| Effort:Reward | 68.064 | 16 | < 0.001 |
| Group:Recipient | 6.407 | 1 | 0.011 |
| Effort:Recipient | 22.855 | 4 | 0.001 |
| Reward:Recipient | 19.966 | 4 | 0.005 |
| Group:Effort:Reward | 33.116 | 16 | 0.007 |
| Group:Effort:Recipient | 23.421 | 4 | 0.001 |
| Group:Reward:Recipient | 2.127 | 4 | 0.712 |
| Effort:Reward:Recipient | 54.743 | 16 | < 0.001 |
| Group:Effort:Reward:Recipient | 21.671 | 16 | 0.462 |

**Linear mixed effects model on force data.** With the force coded as a continuous outcome variable, we defined group, recipient, effort level, reward level, and their interactions as fixed effects. We included a subject-level random intercept.

**
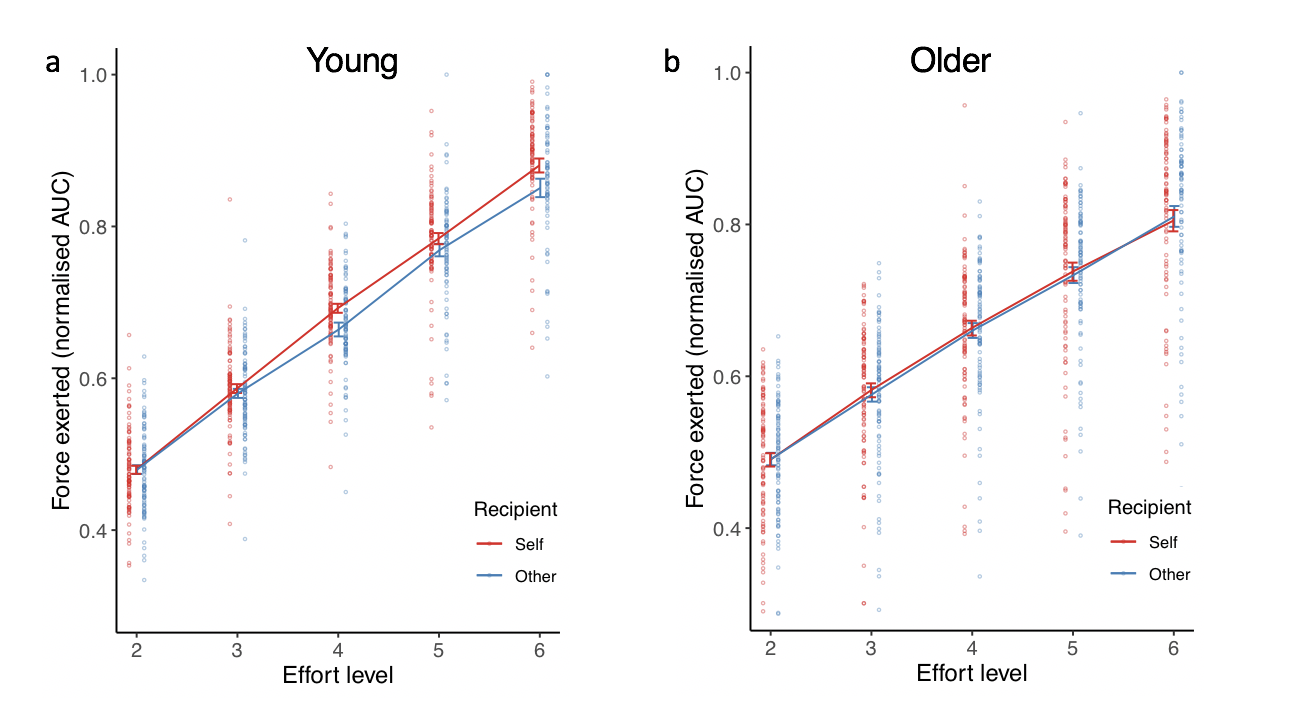
**

**Figure S1. Linked to figure 4. Young adults but not older adults show superficial prosociality.** Panels show the mean area under the curve (AUC) during the 3s force period across effort levels normalised to participants maximum level of force exerted across trials. (a) Replication of Lockwood et al. (Lockwood et al., 2017) showing over-energisation of force at higher effort levels for self compared to other, suggesting ‘superficial prosociality’ – namely even after choosing to help the other person younger adults applied less force than when choosing to help themselves. (b) Older adults showed no difference in amount of force exerted for self and other at any of the effort levels. Overall there was a significant group x recipient x force interaction that reflected these group differences in energisation (*X^2^_(4)_* = 25.956, *p*<.001). Post-hoc comparisons showed a group x recipient interaction was significant at effort levels 4,5 and 6 (all *ps*<.012). Error bars show +/- SEM. For plot displaying all data point see supplementary figure S

**References**

Chen, X., Voets, S., Jenkinson, N., & Galea, J. M. (2019). Dopamine-dependent loss aversion during effort-based decision-making. *The Journal of Neuroscience*, 1760–19. https://doi.org/10.1523/JNEUROSCI.1760-19.2019

Chong, T. T.-J., Apps, M., Giehl, K., Sillence, A., Grima, L. L., & Husain, M. (2017). Neurocomputational mechanisms underlying subjective valuation of effort costs. *PLOS Biology*, *15*(2), e1002598. https://doi.org/10.1371/journal.pbio.1002598

Hartmann, M. N., Hager, O. M., Tobler, P. N., & Kaiser, S. (2013). Parabolic discounting of monetary rewards by physical effort. *Behavioural Processes*, *100*(0), 192–196. http://dx.doi.org/10.1016/j.beproc.2013.09.014

Klein-Flugge, M. C., Kennerley, S. W., Saraiva, A. C., Penny, W. D., & Bestmann, S. (2015). Behavioral modeling of human choices reveals dissociable effects of physical effort and temporal delay on reward devaluation. *PLoS Computational Biology*, *11*(3). https://doi.org/10.1371/journal.pcbi.1004116

Lockwood, P. L., Hamonet, M., Zhang, S. H., Ratnavel, A., Salmony, F. U., Husain, M., & Apps, M. A. J. (2017). Prosocial apathy for helping others when effort is required. *Nature Human Behaviour*, *1*, 0131.
